# Exploring the organismal role of UFMylation in development, stress resilience, and neurological function in *Caenorhabditis elegans*

**DOI:** 10.1016/j.jbc.2026.113247

**Published:** 2026-06-12

**Authors:** Charlotte Sophia Kaiser, Janina Kahl, Emily Jeanne Kouekem, Emma Schröder, Luka Ressmann, Antonio Miranda-Vizuete, Eva Liebau

**Affiliations:** 1 Institute of Integrative Cell Biology and Physiology, University of Münster, Münster, Germany; 2 Redox Homeostasis Group, Instituto de Biomedicina de Sevilla (IBIS), Hospital Universitario Virgen del Rocío/CSIC/Universidad de Sevilla, Sevilla, Spain

**Keywords:** *C. elegans*, ubiquitin-fold modifier 1, UFM1, UFMylation, endoplasmic reticulum stress, neurodegeneration

## Abstract

UFMylation is a posttranslational modification that conjugates ubiquitin-fold modifier 1 to substrate proteins, regulating fundamental processes including ribosomal homeostasis, the endoplasmic reticulum (ER) stress response and DNA damage repair. While loss-of-function mutations in the UFMylation cascade cause lethality in mammals, they are viable in *Caenorhabditis elegans*, offering a unique opportunity to investigate its physiological role at the organismal level. We demonstrate that UFM-1 expression progressively increases from larval stages to adulthood, with predominant localization in intestinal cells. Its expression is upregulated during ER stress and autophagy induction, linking it to these pathways. We used CRISPR/Cas9 to create a targeted *ufm-1* loss-of-function mutant, which revealed that UFMylation is crucial for lifespan, development, and reproduction, with mutants exhibiting increased gonadal dysfunction and sterility. Deletion of *ufm-1* enhanced tolerance to various stressors, a resilience potentially arising from a hormetic response to persistent ER stress. Loss of *ufm-1* selectively activated the unfolded protein response in the ER but not in mitochondria. Notably, *ufm-1* loss exacerbated proteotoxicity in *C. elegans* muscle-expressed models of protein aggregation, accelerating paralysis and increasing the number and size of amyloid-β, α-synuclein, and polyQ aggregates. Furthermore, mutant worms displayed impaired locomotion, including altered swimming patterns resembling those of aging worms, stemming from accelerated, age-dependent sensory neuron dysfunction, and structural neurodegeneration.

Ubiquitin-fold modifier 1 (UFM1) is a ubiquitin-like protein discovered in the early 2000s (1). Like other ubiquitin-like proteins, UFM1 is translated as an inactive precursor, which must be processed before it can conjugate to target proteins *via* an isopeptide bond. This maturation step, carried out by UFM1-specific proteases UfSP1 and UfSP2, involves the cleavage of two amino acids from the C terminus, exposing a glycine residue crucial for subsequent conjugation. After maturation, UFM1 is activated in an ATP-dependent manner mediated by its noncanonical E1 enzyme ubiquitin-like modifier activating enzyme 5 (UBA5). UFM1 is transferred to its E2 enzyme, the UFM1 conjugating enzyme 1 and then covalently bound to target proteins. This is accomplished *via* a UFM1-specific ligating E3 complex, including the UFM1 ligating enzyme 1 and the adapter proteins UFM1 binding protein 1 (UFBP1) and the CDK5 regulatory subunit-associated protein 3 (2, 3, 4, 5).

The UFM1 cascade regulates several key cellular processes including the maintenance of endoplasmic reticulum (ER) homeostasis, facilitation of vesicle trafficking, coordination of autophagy, and regulation of transcription, DNA damage response, and the cell cycle. It also plays a role in hematopoietic cell differentiation and survival (2, 6, 7, 8). Consistent with its important physiological role, disruptions in the UFM1 cascade are associated with a spectrum of diseases. For example, loss of UFMylation in mouse embryos results in severe anemia, demonstrating a critical role of UFMylation in erythropoiesis (9, 10, 11). The UFM1 cascade has also been implicated in metabolic disorders such as diabetes (12, 13) and in various aspects of tumorigenesis, where altered UFMylation may contribute to cancer development and progression (14, 15, 16, 17). Given its role in ER homeostasis, UFMylation is further associated with diseases driven by ER stress, including ischemic heart disease, cardiomyopathy, kidney atrophy, and liver fibrosis (18, 19, 20, 21). Mutations in UFM1 cascade components are also linked to skeletal disorders, such as Beukes hip dysplasia and spondyloepimetaphyseal dysplasia (22, 23, 24). Furthermore, UFM1 plays a role in several neurodevelopmental conditions including autosomal recessive cerebellar ataxia (25), early-onset encephalopathy (26, 27, 28, 29, 30, 31, 32, 33), and hypomyelination with atrophy of the basal ganglia and cerebellum (34).

In previous collaborative studies exploring the role of the UFM1 cascade in severe neurodevelopmental disorders, we demonstrated that biallelic pathogenic variants in *UBA5* impair the enzyme’s catalytic activity, disrupting the formation of the essential UFM1–UBA5 intermediate and the transfer of activated UFM1 to UFM1 conjugating enzyme 1. The resulting clinical severity correlates with residual enzymatic function (26, 28, 31). Compound heterozygous null/hypomorphic alleles cause the most severe presentation, whereas complete loss of UFMylation is embryonic-lethal in mammals. Although these studies established a clear genotype-biochemistry-phenotype link, they were confined to defining the molecular defect.

To understand how these biochemical deficiencies translate into organismal physiology and neurological symptoms, we turned to *Caenorhabditis elegans*. Unlike in mammalian systems, where complete loss of UFMylation is lethal, deletions of core pathway genes are viable in *C. elegans*, providing a unique *in vivo* model to study its role in life-history traits, aging, and stress responses. Our foundational work in this model characterized the evolutionarily conserved UFM1 cascade and revealed that loss-of-function mutants, such as *uba-5(ok3364)* and *ufc-1(tm4888)*, displayed pleiotropic phenotypes including developmental delays, reduced fecundity and shortened lifespan. We further established that the cascade modulates stress resilience, particularly the ER stress response, and that its disruption compromises neurotransmission (26). These findings positioned *C. elegans* as a powerful *in vivo* model for UFMylation but left the central modifier, UFM1 itself, uncharacterized at the organismal level.

Therefore, in this study, we generated a *ufm-1* loss-of-function mutant to define its physiological role. We focused on motility, behavior and stress responsiveness, traits relevant to the neuromuscular and neurological impairments seen in patients. Finally, we investigated the role of UFM-1 in maintaining neuronal integrity during aging and assessed whether UFM-1 influences protein aggregation and toxicity using established *C. elegans* models of proteotoxicity.

## Results

### UFM-1 expression under normal and stress conditions

**Figure 1:**
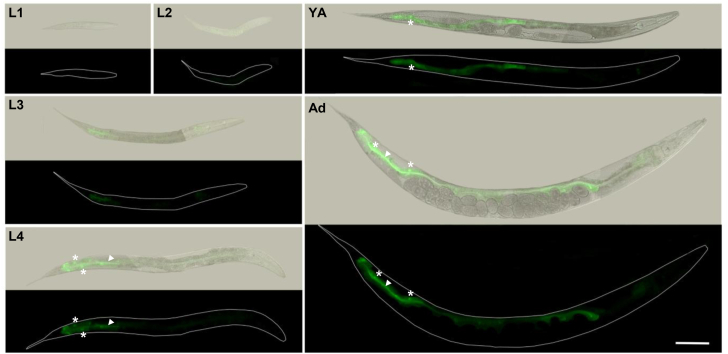
**GFP::UFM-1 expression in *C. elegans* development.** The GFP-tagged UFM-1 protein was expressed under its native promoter to monitor localization and developmental expression. At early larval stages (L1 and L2), GFP::UFM-1 expression was minimal or undetectable. Beginning at the L3 stage, GFP::UFM-1 expression became visible, notably in the intestinal cells, with a stronger signal observed in the posterior region. By the L4 stage and into adulthood, expression intensified and was localized to various cellular compartments within the intestine. Specifically, GFP fluorescence was detected in the cytosol, nuclei (indicated by asterisks) and along the luminal surface of intestinal cells (arrow head). The most prominent GFP signal appeared in the posterior intestine, though expression extended throughout the intestinal tract in adult worms. (L1-4, larval stages 1–4; YA, young adult; Ad, adult; the scale bar represents 100 μm). UFM1, ubiquitin-fold modifier 1.

To gain insight into the function of UFM-1 in *C. elegans*, first a localization study was carried out. To maintain the functionality of the active glycine site of UFM-1 (3), GFP was fused to its N terminus under the control of the native promoter (*ufm-1p::gfp::ufm-1*) and this construct was used to generate transgenic animals. Subsequently, expression and localization were analyzed throughout the different developmental stages of *C. elegans* under standard conditions (Fig. 1). While only a faint to negligible expression of GFP::UFM-1 can be seen in the L1 and L2 larval stages, a GFP signal can be detected in the intestine starting from the L3 stage up to adulthood, with the signal localized in the cytosol and also showing perinuclear or potentially nuclear localization (Fig. S1*A*). High-resolution confocal imaging further supported that the signal is present within intestinal cells and not in the intestinal lumen. To confirm that the observed signal is specific, we performed *ufm-1* RNAi, which resulted in a clear reduction of GFP::UFM-1 fluorescence (Fig. S1*C*).

**Figure 2:**
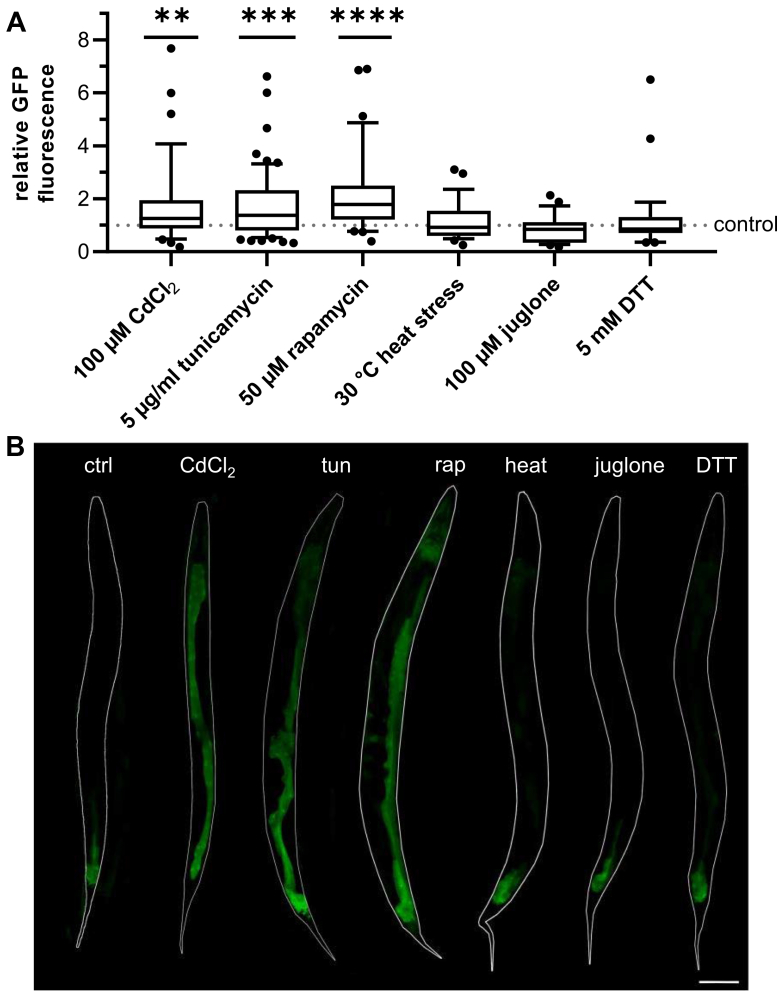
**GFP::UFM-1 expression in *C. elegans* following exposure to various stressors.** To assess the stress-responsive expression of UFM-1, *C. elegans* expressing GFP-tagged UFM-1 under its native promoter were exposed to different stress conditions for 4 h. *A*, fluorescence intensity was analyzed using ImageJ software across three independent trials. Each treatment condition was normalized to untreated controls (n ≥ 30 worms per condition). Statistical significance relative to control was assessed using the Mann-Whitney U test (CdCl_2_*p* = 0.0093; tunicamycin *p* = 0.0005, and rapamycin *p* < 0.0001). Box and whisker plots show the median (*central line*), 25th to 75th percentiles (*boxes*), and 10th to 90th percentiles (*whiskers*). Values outside this range are displayed as individual points. *B*, it shows representative worms of each condition (the scale bar represents 100 μm). Results indicated that exposure to cadmium chloride (CdCl_2_, 100 μM), tunicamycin (tun, 5 μg/ml), and rapamycin (rap, 50 μM) significantly upregulated GFP::UFM-1 expression compared to the control (ctrl), while heat shock (heat, 30 °C), juglone (0.1 mM), and DTT (5 mM) did not yield a statistically significant increase. UFM1, ubiquitin-fold modifier 1.

To determine if UFM-1’s role in stress response extends beyond ER homeostasis, we analyzed its expression following exposure to several compounds that induce distinct types of cellular stress. Specifically, we applied cadmium chloride, a heavy metal that induces oxidative stress and interferes with antioxidant enzymes; tunicamycin, an ER stressor that blocks N-linked glycosylation; and rapamycin, an mTOR inhibitor that induces metabolic and autophagic stress. We found that exposure to these compounds upregulated GFP::UFM-1 expression. This increase was predominantly localized to the intestine, a key defensive tissue in *C. elegans* responsible for detoxification and stress signaling. In contrast, heat shock, a broad physical stressor; juglone, a redox-cycling compound that generates superoxide and hydrogen peroxide while also inhibiting electron transport and various enzymes; and DTT, a reducing agent that disrupts disulfide bonds, did not alter reporter expression, indicating that UFM-1 induction is stressor-specific rather than part of a general stress response (Figs. 2 and S2 for concentration-dependent analyses), at least for the compounds tested.

### Phenotypic characterization of *ufm-1* loss of function mutation

To investigate the role of UFM-1 in *C. elegans*, we generated a complete loss of function allele using CRISPR/Cas9 gene editing. This was essential, as the only available deletion allele affects both *ufm-1* and the adjacent gene *coq-5* and is homozygous lethal, preventing analysis of *ufm-1*-specific phenotypes in adult animals (https://cgc.umn.edu/strain/VC925). We designed a single-guide RNA (sgRNA) targeting the 5′ end of the *ufm-1* gene (Fig. S3) and employed the *dpy-10* coconversion strategy for efficient screening (35). A repair template was engineered to introduce a precise 21 bp sequence immediately after the start codon. This insertion introduces stop codons in all three reading frames to ensure complete translational termination and incorporates a diagnostic restriction site for genotyping (Fig. S3*A*). Homozygous mutants were isolated in the F2 generation and confirmed by restriction analysis, sequencing and Western blot analysis using a rabbit polyclonal antibody against UFM-1 (Fig. S2, *B* and *C*). The strain was outcrossed three times to minimize background mutations, establishing the *ufm-1* KO line (WWU1002 *(ufm-1(eva202)III)*) for phenotypic analysis.

**Figure 3:**
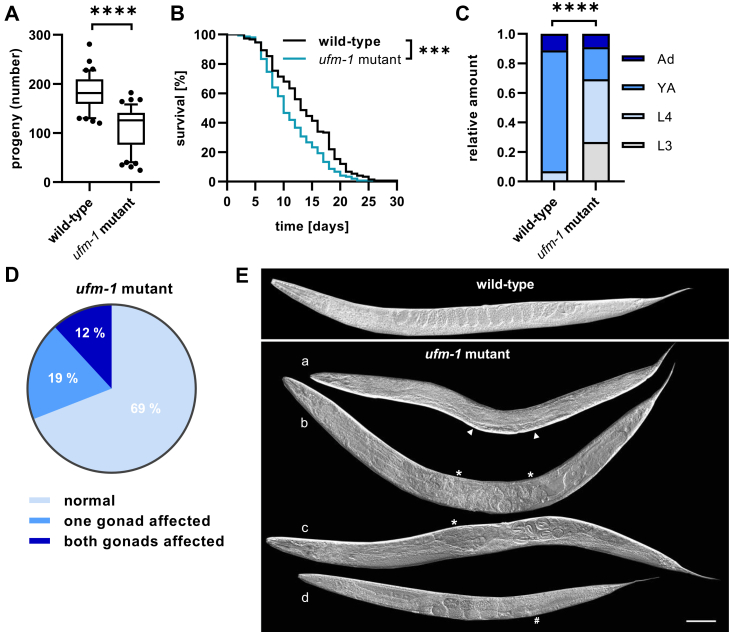
**Life history traits of *C. elegans* wild-type and *ufm-1* loss-of-function mutants.***A*, brood size was measured by determining the total number of progeny produced by wild-type (*wt*, n = 25) and *ufm-1* deletion mutant (*ufm-1* mutant, n = 25) strains, with statistical comparison using a Mann–Whitney U test (*p* < 0.0001). *Box* and *whisker* plots show the median (central line), 25th to 75th percentiles (*boxes*), and 10th to 90th percentiles (*whiskers*). Values outside this range are displayed as individual points. *B*, lifespan was evaluated using Kaplan-Meier survival analysis (Log-Rank test) for both strains (*wt*, N = 3, n = 150; *ufm-1* mutant, N = 3, n = 150 (*p* < 0.0001)). *C*, larval development of *ufm-1* mutants was compared to wild-type. Larval stages (L3-4, larval stage 3–4; YA, young adult; Ad, adult) were counted at 24 h, 48 h (Figs. S3), and 72 h (*wt*, n = 254; *ufm-1* mutant, n = 109 (*p* < 0.0001), Sidak’s test). *D*, morphological analysis of *ufm-1* mutants. The pie chart illustrates that the *ufm-1* mutants had a significantly higher number of malformed gonads compared to wild-type (*wt*, n = 136; *ufm-1* mutant, n = 195). *E*, compared to wild-type (*upper panel*), the *ufm-1* deletion mutants (*lower panel*) showed morphological differences, particularly in gonads and embryo development. These phenotypes ranged from the complete absence of gonads (a, highlighted by an *arrow head*) to malfunctions in embryogenesis (b-d). One phenotype displayed *egg-shaped structures* lacking typical proliferation stages of embryonic development (*asterisks*). This occurred either in both gonad arms (b) or in only one gonad arm, while the other appeared to have normal embryonic development (c). Other phenotypes showed two functional gonads, with one displaying delayed development and a reduced number of embryos (d, affected arm indicated by a hashtag) (the scale bar represents 100 μm). UFM1, ubiquitin-fold modifier 1.

This *ufm-1* loss-of-function mutant was analyzed for key metrics such as reproduction, lifespan, and larval development. The mutants exhibited a significantly reduced reproductive output (WT: 184 ± 35 offspring; *ufm-1* mutant: 108 ± 42 offspring) and a shortened lifespan (Fig. 3, *A* and *B*). Developmental progression through larval stages was assessed at 24, 48, and 72 h. At 24 and 48 h, the *ufm-1* deletion mutants (n = 142) showed development comparable to WT animals (n = 283) (Fig. S4). However, by 72 h, a noticeable developmental delay was observed in the *ufm-1* mutants (Fig. 3*C*). These results demonstrate that *ufm-1* is crucial for normal reproductive output and developmental progression in *C. elegans*, emphasizing its role in key life history traits.

Consistent to their reduced brood size, *ufm-1* mutants exhibited a range of gonadal and embryonic abnormalities (Fig. 3, *D* and *E*). In the most severe cases, both gonad arms were entirely absent; in others, one arm was noticeably shortened. By contrast, some mutants retained two normally U-shaped arms, yet still produced embryos that arrested or developed more slowly. Quantitatively, 69% of *ufm-1* mutants formed both gonad arms, 20% displayed a single truncated arm, and 11% showed complete bilateral agenesis. This spectrum of phenotypes highlights the variable but penetrant requirement for UFM-1 in gonad morphogenesis.

### Stress resistance of *ufm-1* loss-of-function mutants under heavy metal, osmotic, oxidative, and ER stress

**Figure 4:**
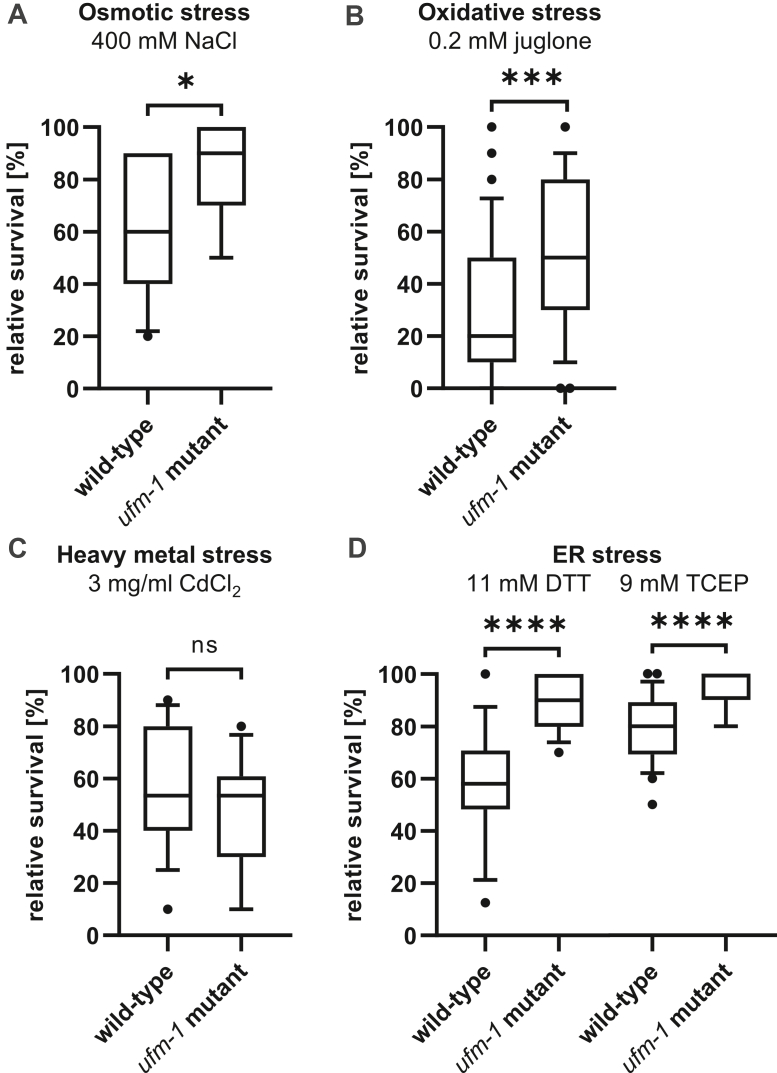
**Survival assays of *ufm-1* mutant under various stressors.** The stress resistance of *C. elegans ufm-1* mutant was assessed by comparing their survival rates to wild-type worms (*wt*) under different conditions. Survival was evaluated after incubation with: (*A*) 400 mM NaCl for 24 h (*wt*, n = 100; *ufm-1* mutant, n = 180 (*p* = 0.0134)), (*B*) 0.2 mM juglone for 18 h (*wt*, n = 390; *ufm-1* mutant, n = 390 (*p* = 0.0004)), (*C*) 3 mg/ml CdCl_2_ for 18 h (*wt*, n = 100; *ufm-1* mutant, n = 100), and (*D*) 11 mM DTT for 18 h (*wt*, n = 140; *ufm-1* mutant, n = 140 (*p* < 0.0001)) or 9 mM TCEP for 24 h (*wt*, n = 220; *ufm-1* mutant, n = 210 (*p* < 0.0001)) (Mann–Whitney U test). Box and whisker plots show the median (*central line*), 25th to 75th percentiles (*boxes*), and 10th to 90th percentiles (*whiskers*). Values outside this range are displayed as individual points. TCEP, tris(2-carboxyethyl)phosphine; UFM1, ubiquitin-fold modifier 1.

The stressor-specific upregulation of GFP::UFM-1 suggested its activity might be functionally important for survival. To test this, we assessed stress resistance in the *ufm-1* loss-of-function mutant by exposure to various stressors (Fig. 4). Notably, the mutant exhibited enhanced resistance to osmotic, oxidative, and ER stress compared to WT controls, indicating a potential adaptive response that confers resistance to specific insults at the cost of baseline fitness. However, the deletion mutant did not show increased resistance to heavy metal stress, suggesting that *ufm-1* modulates stress pathways selectively, with a role more closely linked to osmotic stress, oxidative damage, and ER homeostasis.

### Assessment of ER- and mitochondrial-unfolded protein response (UPR_ER_ and UPR_mito_)

To monitor the activation of cellular stress responses upon *ufm-1* depletion, we utilized well-established *C. elegans* transcriptional reporters for the unfolded protein response. The *hsp-4::gfp* reporter, where *hsp-4* is one of two *C. elegans* BiP/GRP78 orthologs, specifically indicates activation of the UPR_ER_. For comparison, and to assess the specificity of the stress response, we also employed the *hsp-6::gfp* reporter, which is induced specifically upon UPR_mito_. This allowed us to determine whether loss of UFM-1 triggers a compartment-specific proteostatic disruption.

**Figure 5:**
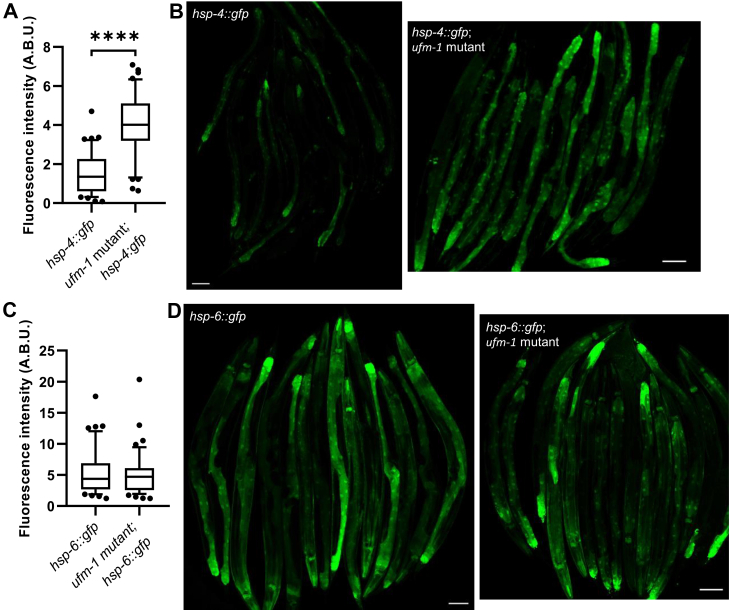
***ufm-1* knockout selectively activates UPRE_R_ but not UPR_mito_.***A*, quantification of fluorescence intensity shows that *ufm-1* deletion significantly induces the UPR_ER_ reporter *hsp-4::gfp* compared to the control (*hsp-4::gfp*, n = 42; *ufm-1*; *hsp-4::gfp*, n = 46; Mann-Whitney U test (*p* < 0.0001)). *B*, representative fluorescence images of *hsp-4::gfp* worms (*left*) or *ufm-1*; *hsp-4::gfp* (*right*) (the scale bar represents 100 μm). *C*, quantification shows that *ufm-1* deletion does not alter the expression of the UPR_mito_ reporter *hsp-6::gfp* (*hsp-6::gfp*, n = 42; *ufm-1*; *hsp-6::gfp*, n = 45; Mann-Whitney U test). *D*, representative fluorescence images of *hsp-6::gfp* worms (*left*) or *ufm-1*; *hsp-6::gfp* (*right*) (the scale bar represents 100 μm). *Box* and *whisker* plots show the median (*central line*), 25th to 75th percentiles (*boxes*), and 10th to 90th percentiles (*whiskers*). Values outside this range are displayed as individual points. UFM1, ubiquitin-fold modifier 1.

We generated double mutants carrying the *ufm-1* null allele (*eva202*) together with either the *hsp-4::gfp* or *hsp-6::gfp* reporter. Because *ufm-1* maps to chromosome III and both reporters map to chromosome V, we successfully obtained *ufm-1; hsp-4::gfp* and *ufm-1; hsp-6::gfp* double mutants by genetic crossing. Young adult worms of each genotype were imaged under identical conditions to quantify whole-animal GFP intensity (Fig. 5). Loss of *ufm-1* in the double mutants led to a significant increase in fluorescence in the *ufm-1; hsp-4::gfp* strain compared to the control, confirming activation of the UPR_ER_ (Fig. 5, *A* and *B*). In contrast, the *ufm-1; hsp-6::gfp* strain showed no change, demonstrating that loss of UFM-1 does not trigger UPR_mito_ (Fig. 5, *C* and *D*). These results indicate that *ufm-1* depletion selectively induces ER stress signaling without engaging mitochondrial proteostasis pathways.

### Analysis of locomotion in the *ufm-1* KO mutant

Given the association between UFM1 cascade mutations and human neurological disorders, which often involve motor and sensory deficits, we initially performed chemotaxis assays toward the attractant diacetyl (36). However, even after extending the incubation period to account for reduced motility, the pronounced locomotion deficits of the ufm-1 mutant made it difficult to distinguish between impaired sensory perception and compromised motor output. Therefore, we focused our quantitative analysis on locomotor behavior.

**Figure 6:**
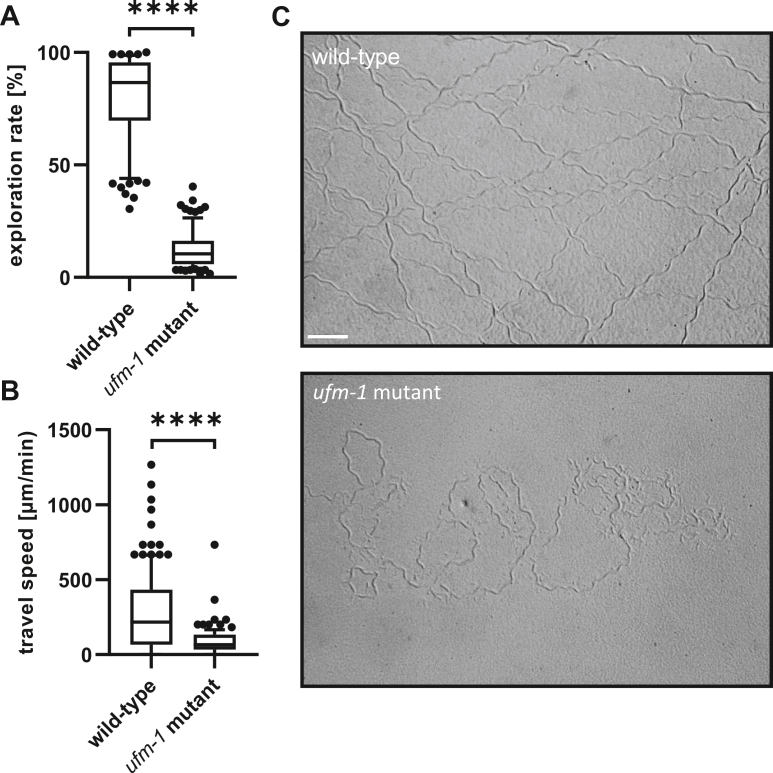
**Behavioral parameters of *ufm-1* loss-of-function mutants compared to wild-type worms on solid media.***A*, exploration behavior is significantly reduced in *ufm-1* mutant compared to wild-type (*wt*, n = 90; *ufm-1* mutant, n = 88; Mann-Whitney U test (*p* < 0.0001)). *B*, radial locomotion assay shows a slower travel speed in *ufm-1* mutant (*wt*, n = 130; *ufm-1* mutant, n = 100; Mann-Whitney U test (*p* < 0.0001)). *Box* and *whisker* plots show the median (*central line*), 25th to 75th percentiles (*boxes*), and 10th to 90th percentiles (*whiskers*). Values outside this range are displayed as individual points. *C*, tracks of the *ufm-1* deletion mutant lack the typical sinusoidal waveform seen in wild-type worms, exhibiting reduced spontaneous movement and branching patterns (the scale bar represents 1 mm). UFM1, ubiquitin-fold modifier 1.

Deletion of *ufm-1* in *C. elegans* resulted in significant motility impairments, including a reduced exploration rate (13% of plate coverage compared to 79% in WT) (Fig. 6, *A* and *C*) and slower radial locomotion speed (95 μm/min *versus* 290 μm/min in WT) (Fig. 6*B*). These motility defects point to neuromuscular impairments and changes in movement patterns often seen in *C. elegans* mutants that affect motor function or nerve signaling (37).

The CeleST software (38) provides a precise, quantitative method for assessing locomotor dysfunction in *C. elegans* mutants by tracking detailed movement parameters. Using CeleST, we analyzed swimming behavior of the *ufm-1* deletion mutant in liquid. Compared to WT, *ufm-1* mutant showed a reduced wave initiation rate (Fig. S5*A*), an increased number of body waves (Fig. S5*B*), greater body stretch (Fig. S5*C*), decreased travel speed (Fig. S5*D*), lower brush-stroke frequency (Fig. S5*E*), and a diminished overall activity index (Fig. S5*F*). These observations point to a disruption in motor coordination and align with defects seen in solid media assays. Despite pronounced swimming impairments, *ufm-1* deletion animals retain the ability to swim and can transition from crawling to swimming, suggesting that the core regulatory mechanisms underlying these two modes of locomotion remain largely intact in the absence of UFM-1.

To validate that the observed phenotypes were specifically due to the loss of *ufm-1*, we generated a rescue strain (WWU1018) by expressing a *ufm-1p::ufm-1* transgene in the *ufm-1(eva202)* deletion mutant. Western blot analysis confirmed the expression of UFM-1 protein in this strain (Fig. S3*C*). This rescue strain was used to assess all phenotypes, including motility, brood size, lifespan, development, and DTT stress resistance. Expression of UFM-1 partially rescued the deletion phenotypes, ameliorating defects in motility, and DTT stress resistance (Fig. S6, *A*, *B*, and *F*). The rescued strain exhibited a lifespan equivalent to WT (Fig. S6*D*). While the extrachromosomal array carrying *ufm-1* driven by its own endogenous promoter partially restored development after 72 h (Fig. S6*E*), it did not restore brood size; both the *ufm-1* KO and array-carrying worms showed similarly reduced reproduction (Fig. S6*C*). This could be due to extrachromosomal arrays being silenced in the germline. Development, by contrast, can be rescued by somatic expression alone. These results confirm that the observed physiological defects are a direct consequence of the loss of *ufm-1*.

### Role of UFM-1 in neurodegeneration and proteotoxicity

**Figure 7:**
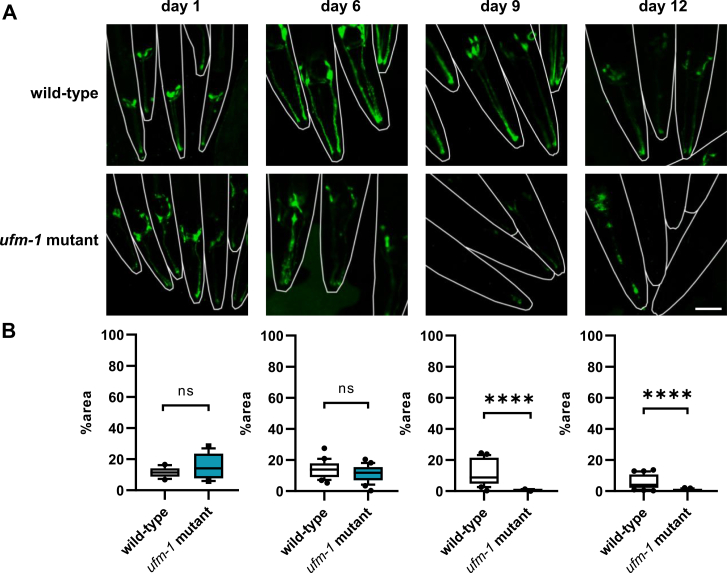
**Loss of UFM-1 accelerates age-associated neuronal decline.***A*, dye-filling of amphid neurons with DiO in wild-type and *ufm-1* deletion mutants at different days of adulthood. The *ufm-1* mutant exhibits a more severe, age-dependent reduction in dye uptake, indicating progressive sensory neuron dysfunction (the scale bar represents 50 μm). *B*, dye uptake was quantified using ImageJ to analyze fluorescent area per head region (day 1 *wt*, n = 14; *ufm-1* mutant, n = 17; Mann-Whitney U test, day 6 *wt*, n = 26; *ufm-1* mutant, n = 24; Mann-Whitney U test, day 9 *wt*, n = 28; *ufm-1* mutant, n = 12; Mann-Whitney U test *(p < 0.0001)*, day 12 *wt*, n = 33; *ufm-1* mutant, n = 27; Mann-Whitney U test *(p < 0.0001)*) Box and whisker plots show the median (*central line*), 25th to 75th percentiles (*boxes*), and 10th to 90th percentiles (*whiskers*). Values outside this range are displayed as individual points. UFM1, ubiquitin-fold modifier 1.

The pronounced impact of *ufm-1* loss on motility and chemotactic behavior suggested underlying neurological dysfunctions. To investigate this, we assessed neuronal health and integrity in *ufm-1* deletion mutants during aging. First, we used a functional dye-filling assay, where uptake of the fluorescent dye DiO by the sensory amphid neurons serves as a readout of neuronal function and ciliary integrity. Although dye staining was similar in young adults of both strains, the *ufm-1* mutant exhibited a more pronounced age-dependent decrease in dye uptake compared to the WT (Fig. 7), indicating an accelerated loss of sensory neuron function.

**Figure 8:**
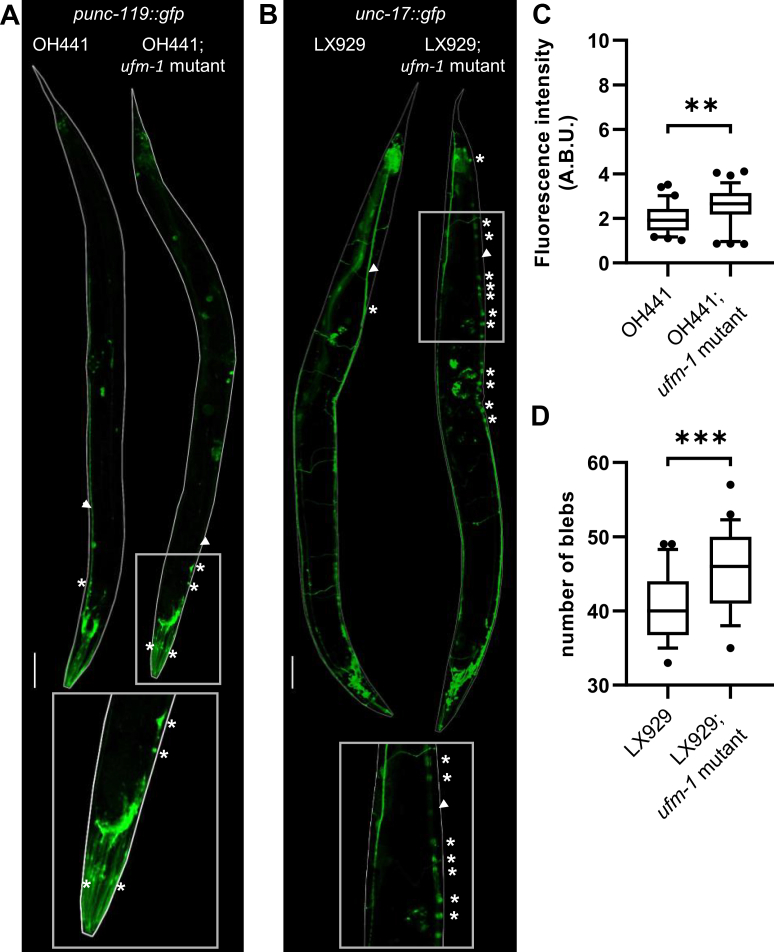
**Loss of UFM-1 accelerates neuronal decline in pan-neuronal and cholinergic reporter strains.** Representative images of (*A*) pan-neuronal GFP signal (OH441, *otls45[unc-119::gfp*) and of (*B*) cholinergic neuron-specific GFP signal (LX929, *vsIs48 [unc-17::gfp]*) in wild-type and in the *ufm-1* mutant background at days 6 of adulthood (the scale bar represents 100 μm). The *ufm-1* mutant shows increased age-associated neurite fragmentation (*arrowheads*) and a decline in overall structural integrity as beading and swollen cell bodies (asterisks) compared to wild-type. *C*, overall GFP intensity is significantly increased at 6 days after deletion of *ufm-1* in pan-neuronal reporter strain (OH441 n = 30; OH441; *ufm-1* n = 36 (*p* = 0.0027); Mann-Whitney U test). *D*, number of blebs per worm is increased at 6 days after deletion of *ufm-1* in cholinergic reporter strain (LX929 n = 28; LX929; *ufm-1* n = 28 (*p* = 0.0002); unpaired *t* test). *Box* and *whisker* plots show the median (*central line*), 25th to 75th percentiles (*boxes*), and 10th to 90th percentiles (*whiskers*). Values outside this range are displayed as individual points. UFM1, ubiquitin-fold modifier 1.

We next assessed neuronal morphology by introducing the *ufm-1* deletion into a pan-neuronal reporter strain (OH441, *otls45[unc-119::gfp])* (Fig. 8*A*) and a cholinergic reporter strain (LX929, *vsIs48 [unc-17::gfp]*) (Fig. 8*B*). With age, the *ufm-1* mutants displayed more severe signs of neurodegeneration, such as swollen axon cell bodies, compared to controls (Fig. 8, *C* and *D*). Together, these findings demonstrate that the loss of *ufm-1* compromises both the function and structural integrity of neurons during normal aging.

**Figure 9:**
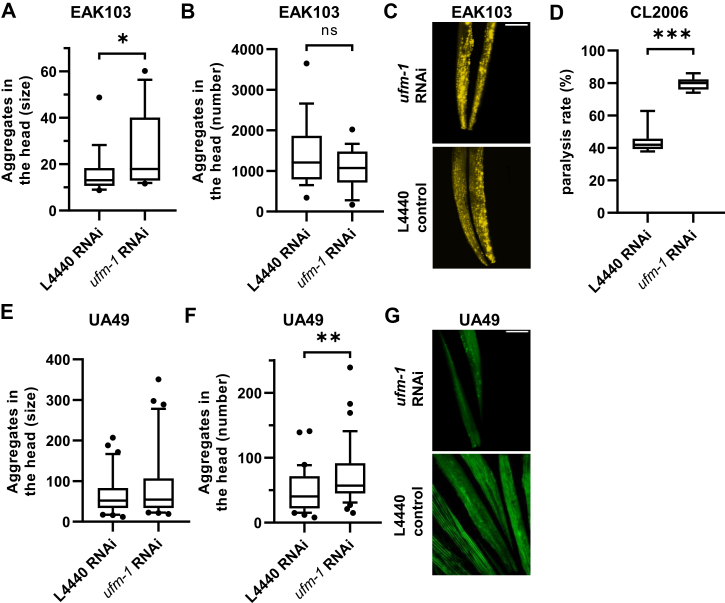
**UFM-1 modulates proteotoxicity and aggregation in *C. elegans* neurodegeneration models.** The effect of *ufm-1* knockdown by RNAi was assessed in three established models. *A*, quantification of aggregate size (*p* = 0.0287) and (*B*), aggregate number in a body-wall muscle-expressed, GFP-tagged polyQ model (strain EAK103) (L4440 control, n = 17; *ufm-1* RNAi, n = 17; Mann-Whitney U test). *C*, representative images of polyQ aggregates in the head region of *C. elegans* from the experiment in (*A*) and (*B*). *D*, paralysis assay in an Aβ3-42 expression model (strain CL2006) comparing control (L4440) and *ufm-1* knockdown conditions (L4440, n = 80; *ufm-1* RNAi, n = 80 (*p* = 0.0002); Mann-Whitney U test). *E*, quantification of aggregate size and *F*, aggregate number (*p* = 0.0049) in a body-wall muscle-expressed, GFP-tagged α-synuclein model (strain UA49) (L4440 control, n = 32; *ufm-1* RNAi, n = 33; Mann-Whitney U test). *Box* and *whisker* plots show the median (*central line*), 25th to 75th percentiles (*boxes*), and 10th to 90th percentiles (*whiskers*). Values outside this range are displayed as individual points. *G*, representative images of α-synuclein aggregates in the head region from the experiment in (*E*) and (*F*). UFM1, ubiquitin-fold modifier 1.

We next asked whether the compromised neuronal health in *ufm-1* mutants would heighten sensitivity to disease-associated proteotoxicity, the cellular damage caused by misfolded or aggregated proteins. To test this, we used well-established *C. elegans* models of proteotoxicity (Fig. 9).

In a muscle-specific GFP-tagged polyQ model of Huntington’s disease *ufm-1* knockdown significantly increased the size of protein aggregates without affecting their number (Fig. 9, *A*–*C*).

In a second model of β-amyloid toxicity (strain CL2006), RNAi-mediated downregulation of *ufm-1* significantly increased paralysis rates compared to controls (Fig. 9*D*), suggesting that UFM-1 mitigates β-amyloid-induced toxicity.

Finally, in a model of α-synuclein aggregation (strain UA49), *ufm-1* knockdown did not alter aggregate size (Fig. 8*E*) but significantly increased their number (Fig. 9, *F* and *G*).

Collectively, these results indicate that UFM-1 modulates proteostasis in a context-dependent manner, influencing the aggregation dynamics of diverse neurodegeneration-associated proteins and underscoring its critical role in maintaining proteostasis.

## Discussion

The UFM1 cascade has emerged as a key regulator of cellular homeostasis (5, 39). Using *C. elegans* as a model, this study provides novel insights into the physiological role of UFMylation at the organismal level, particularly in development, stress resilience, and neuromuscular function. Although our findings reveal that *ufm-1* is dispensable for viability in *C. elegans*, it is essential for maintaining normal lifespan, reproduction, and neuromuscular function, highlighting its relevance to human diseases linked to UFM1 cascade dysfunction. Moreover, our study reveals that UFMylation modulates protein aggregation and neurodegeneration, underscoring its importance in cellular and organismal homeostasis.

### UFM-1 is important for development and reproduction

To determine the role of UFM-1 in *C. elegans*, expression and localization studies were performed using a translational UFM-1::GFP reporter. Under physiological conditions, GFP fluorescence was primarily detected at the posterior end of the intestine, with additional expression observed in rectal cells. In early larval stages, UFM-1 expression was minimal; however, it increased noticeably from the L3 stage onward, showing cytosolic and apical domain localization in intestinal cells. The signal also showed overlap with 4′,6-diamidino-2-phenylindole (DAPI), but this colocalization is not unambiguous evidence for true nuclear localization. Expression peaked during the L4 stage and adulthood. In adult *C. elegans*, several other members of the UFM-1 cascade also displayed predominantly intestinal localization, suggesting a conserved role of this pathway in intestinal function (40).

Interestingly, the UFMylation cascade is highly increased in endocrine secretory cells (12). In mammalian systems, two key components of the UFM1 E3 ligase have been found to be highly expressed in intestinal exocrine secretory cells, including Paneth and goblet cells. At the cellular and molecular levels, deletion of UFM1 ligating enzyme 1 and *ufbp1* resulted in elevated ER stress, activation of the UPR, and the initiation of a cell death program. These results identify the UFMylation pathway as a novel molecular mechanism for controlling homeostasis (41).

The observed spatial and temporal expression pattern is consistent with a possible association of UFM-1 with intestinal cells that have high secretory activity, but we did not directly assess any secretory function. The *C. elegans* intestine is highly active in endocytosis, exocytosis, and intracellular trafficking. Digestive and protective molecules are secreted apically into the intestinal lumen, while nutrients are absorbed *via* endocytosis or specific transporters (42). The apical enrichment of UFM-1 may reflect a role in ER-related processes, though direct functional studies are required to establish this. At the basolateral side, synthesized yolk proteins are exported to provision eggs and waste or signaling molecules are reimported. This dynamic activity is supported by an abundance of stacked rough ER, characteristic of cells with high secretory demands (43). Thus, while our localization data are suggestive, direct secretion assays are needed to determine whether UFM-1 supports intestinal secretory functions.

DAPI colocalization suggests nuclear association, but additional experiments are required to determine whether UFM-1 has any nuclear functions in *C. elegans*. In mammals, UFMylation components have been reported to localize to the nucleus, where they contribute to genome stability by promoting telomere maintenance and modifying key proteins involved in DNA repair, particularly double-strand break repair (5, 44). In addition, UFMylation regulates transcription factor activity, influencing various gene expression patterns (45).

CRISPR/Cas9-mediated deletion of *ufm-1* in *C. elegans* resulted in a range of severe phenotypes, including reduced lifespan, developmental delays, reduced fecundity and distinct gonadal abnormalities, emphasizing the essential role of UFM-1 in organismal development. Although mutants of other components in the UFM-1 cascade, such as the activating enzyme *uba-5(ok3364)* and the conjugating enzyme *ufc-1*(tm4888), also exhibited reduced lifespan and brood sizes, they did not display clear morphological defects (40). In contrast, *ufm-1* mutants showed markedly more severe reproductive impairments, including gonadal malformations, uterine defects, and cases of sterility due to embryonic arrest or disrupted germline architecture.

The fact that *C. elegans* survives without UFM-1 allowed us to study these essential functions in a living organism. In mammals, the essentiality of the UFM1 pathway is underscored by the fact that global KOs of cascade members lead to embryonic lethality, accompanied by hematopoietic failure and liver hypoplasia (9, 11, 46). Though *C. elegans ufm-1* mutants remain viable and capable of producing offspring, their strongly reduced reproductive output and developmental defects parallel the vital role of UFMylation observed across metazoans. These defects align with the notion that UFMylation is critical for cellular homeostasis and normal organogenesis, as has been reported in other metazoan systems (3, 47).

### UFM-1 influences the response to stress through the UPR_ER_

Robust induction of UFM-1 promoter-driven GFP expression was observed following treatment with tunicamycin, rapamycin, and cadmium. Tunicamycin inhibits N-glycosylation, leading to accumulation of misfolded proteins within the ER and activation of the UPR (48). Rapamycin, an inhibitor of the mTORC1 complex, enhances UFM1 expression, facilitating the UFMylation of various substrates involved in autophagic processes. In addition to activating general autophagy, rapamycin promotes ER-phagy, a selective degradation of ER components, which aligns with the proposed role of UFM1 in maintaining ER proteostasis through targeted degradation and remodeling of ER components (49, 50, 51). Cadmium, a toxic heavy metal, also significantly upregulated UFM-1, consistent with its ability to increase reactive oxygen species and specifically generate superoxide and hydrogen peroxide and lipid peroxidation, thereby activating both ER stress pathways and autophagy (52). Unlike juglone, which generates superoxide *via* redox cycling while also inhibiting respiratory enzymes and various other cellular processes, cadmium disrupts multiple antioxidant systems (*e.g.* glutathione depletion) and induces more sustained oxidative stress, which may explain differential UFM-1 responses between these two stressors. In *C. elegans*, cadmium was also shown to activate the *hsp-4::gfp* reporter indicating that it causes ER stress (53).

In contrast, treatment with DTT, which induces ER stress by disrupting disulfide bond formation and altering the ER redox environment, resulted in no observable increase in UFM-1 expression. However, DTT is known to affect not only ER redox balance but also global cellular redox state, mitochondrial function, and protein folding in other compartments (54). Therefore, the absence of UFM-1 induction following DTT treatment could theoretically arise from opposing effects of these additional processes, or from a dilution of ER-specific stress signals by broader cellular perturbations. Nevertheless, the observed difference between DTT and tunicamycin, both canonical ER stressors, suggests that not all forms of ER stress equally activate the UFMylation pathway, potentially pointing to stress-specific thresholds or qualitative differences in UPR activation that may influence UFM-1 regulation. Furthermore, other stressors such as heat shock or the redox cycling agent juglone also failed to significantly elevate UFM-1 levels. This pattern supports the hypothesis that UFM1 activation is selectively responsive to particular classes of ER and metabolic stress, especially those involving glycosylation inhibition and autophagy induction, rather than functioning as a generalized stress response factor.

Our survival assays demonstrate that *ufm-1* deletion mutants are more resistant than WT to osmotic-, oxidative-, and ER-stressors, yet show no protection under cadmium challenge. An enhanced stress tolerance toward pathogenic-, heat-, oxidative-, and ER-stress was also observed in *uba-5(ok3364)* and *ufc-1*(tm4888) mutants where the survival in the presence of cadmium was also reduced (40).

The enhanced stress tolerance observed in the *ufm-1* deletion mutant likely stems from the constitutive activation of the ER UPR, as demonstrated by the upregulation of the *hsp-4::gfp* reporter. HSP-4 is one of two*C. elegans* BiP/GRP78 orthologs, together with HSP-3. Similar findings were reported for the *uba-5(ok3364)* mutant. When crossed with the *hsp-4::gfp* reporter strain (SJ4005), these mutants also showed elevated GFP expression, indicating activation of ER stress pathways in the absence of UFMylation (40). In mammals, the UFM1 pathway deficiency triggers ER stress. Loss of UFBP1 (DDRGK1) increases Xbp1 mRNA splicing and eIF2α phosphorylation, with UFBP1-deficient mice showing impaired hematopoietic stem cell survival due to unresolved ER stress and apoptosis (9). The UFM1 system also protects pancreatic β-cells by maintaining ER homeostasis and insulin secretion and in macrophages, it limits ER stress-induced apoptosis by suppressing proapoptotic UPR signaling (13).

The absence of mitochondrial UPR (*hsp-6::gfp*) activation in *ufm-1* RNAi-treated worms is consistent with the current understanding of UFM1′s role in ER-associated proteostasis, rather than mitochondrial stress pathways that are primarily activated by the accumulation of unfolded or misfolded proteins within the mitochondrial matrix (55).

Only a few studies suggest a potential involvement of UFM1 in mitochondrial homeostasis. In *Leishmania donovani*, components of the UFM1 cascade localize to mitochondria and regulate fatty acid metabolism, impacting energy production and parasite viability (56). Similarly, *uba5* KO in zebrafish causes mitochondrial abnormalities and signs of mitophagy activation (57). Moreover, UFMylation of P4HB, a protein found in both the ER and mitochondria, is essential for mitochondrial function (58).

### UFM-1 is necessary for proper locomotion

*ufm-1* mutants exhibit drastically reduced exploration rates and slower radial locomotion on solid media, consistent with impaired motor function. Liquid-based locomotion analysis using CeleST revealed swimming deficits, including a reduced wave initiation rate, increased body curvature and lower travel speed.

Comparable phenotypes have been observed in other model organisms lacking functional UFM1 pathway components. In *Drosophila*, knockdown of the UFM1-activating enzyme UBA5 resulted in reduced locomotor activity, shorter lifespan, and fewer but enlarged boutons at the neuromuscular junction, indicating abnormal synaptic formation and transmission. Adult flies also display persistent vertical-turned wing postures that fail to unfold at rest and both climbing and flight are severely impaired (25). In zebrafish, *uba5* silencing leads to decreased motility and induces seizure-like movements (26, 57). Finally, human UBA5 mutations cause neurodevelopmental disorders characterized by early-onset encephalopathy and severe motor deficits (26, 29, 30), mirroring the locomotor phenotypes observed in these animal models.

In a mouse model of ALS exhibiting rapid muscle atrophy due to an ALS-causing mutation in the superoxide dismutase 1 gene (59), UFMylation is significantly upregulated. Strikingly, *in vivo* knockdown of UFMylation rescued muscle contraction force, demonstrating its role as a critical regulator of skeletal muscle function (60). Furthermore, using a novel antibody-based enrichment approach to identify UFMylated proteins, a distinct UFMylation signature was observed in human skeletal muscle associated with ALS, including prominent increases in myosin UFMylation (61).

However, the interdependence of sensory perception and motor execution complicates interpretation. UFMylation may act at multiple levels of the nervous system, from sensory detection to downstream signaling and motor output. Dissecting the specific role of UFMylation will require targeted strategies to separate its effects on sensory input from those on motor output.

### UFM-1 deficiency potentiates neurodegenerative phenotypes in *C. elegans*

The UFM1 cascade plays a critical role in brain development and its dysfunction is linked to severe neurodevelopmental disorders such as early-onset encephalopathy (3, 5). Its role in neurodegeneration and proteostasis during protein aggregation, however, is not well defined.

We therefore examined transgenic *C. elegans* expressing Aβ, α-synuclein, or polyQ-Huntingtin in body wall muscles. Following *ufm-1* depletion *via* RNAi, we observed accelerated paralysis in Aβ worms, increased α-synuclein aggregation and enhanced polyQ-Huntingtin aggregate size. These results indicate that UFM1 is involved in maintaining proteostasis in the context of aggregation prone proteins, suggesting UFM1 facilitates the clearance of such proteins.

Recent studies indicate that UFMylation promotes α-synuclein secretion *via* the misfolding-associated protein secretion pathway, potentially influencing its seeding and propagation in Parkinson’s disease. Here, mono-UFMylation acts as a molecular triage signal designating misfolded proteins for unconventional secretion rather than targeting them for proteasomal or lysosomal degradation (62). Furthermore, a CRISPR screen in a human iPSC-derived 4R tauopathy model identified five of six core UFMylation components as top enhancers of Tau propagation. Functional validation showed that knockdown of *UFM1* or *UBA5* reduced Tau spread and Tau protein abundance (63), suggesting UFMylation regulates the stability and propagation of aggregation-prone proteins through unresolved mechanisms.

Furthermore, our analysis extended beyond proteotoxicity in disease models to general neuronal health. We found that a *ufm-1* deletion mutant exhibited an accelerated, age-dependent decline in neuronal integrity, as evidenced by impaired dye-filling in sensory neurons and increased morphological signs of neurodegeneration in pan-neuronal and cholinergic neuron-specific reporter strains (64, 65).

In summary, our study establishes UFM1 as a crucial regulator of organismal physiology in *C. elegans*. We demonstrate its essential role in development and reproduction, its function in modulating stress resilience *via* the UPR_ER_ and its importance for maintaining neuromuscular function and sensory integrity. The increased proteotoxicity observed in the *C. elegans* muscle-based aggregation models, combined with the accelerated neuronal aging, suggest that the UFM1 cascade could be a potential therapeutic target for disorders involving protein misfolding and ER stress. This is further supported by emerging evidence that UFMylation is critical for the peripheral nervous system and muscle homeostasis, with UBA5-deficient zebrafish showing peripheral nerve damage and skeletal muscle mitochondrial dysfunction (57). The notable similarities between our findings and phenotypes in flies, zebrafish and humans with UFMylation deficiencies highlight the evolutionary conservation of this pathway. They also confirm the value of *C. elegans* as a model for understanding UFM1-related diseases.

## Experimental procedures

### *C. elegans* strains, genetic manipulations, and cultivation

WT *C. elegans* (N2 Bristol), GE24 *(pha-1(e2123)III),* OH441 *(otls45[unc-119::gfp]),* LX929 *(vsIs48 [unc-17::gfp]),* CL2006 *(dvIs2[pCL12(unc-54p::human Aβ) + rol-6(su1006)]),* UA49 (*baIn2[unc-54p::α-syn::gfp) + rol-6(su1006)]),* EAK103 *(eeeIs2[unc-54p::Htt513(Q128)::yfp::unc-45 3′UTR]),* SJ4100 *(zcls13 [hsp-6p::gfp + lin-15(+)]),* and SJ4005 *(zcls4 [hsp-4::gfp]V)* were obtained from the *Caenorhabditis* Genetics Center.

We adhere to the standard *C. elegans* nomenclature, where genes are referred to with lowercase, hyphenated names (*e.g.*, *ufm-1*) and their protein products are designated by the corresponding uppercase symbol (*e.g.*, UFM-1).

For expression studies, the translational reporter strain WWU1001 *(evaEx201[ufm-1p::gfp::ufm-1 + pha-1(+)])* was generated by coinjecting the plasmid pPD95_77, which contains the *ufm-1* promoter and *ufm-1* gene, along with pBX as a selection marker, into the GE24 strain. This method facilitates the expression of GFP under the control of the *ufm-1* promoter and allows selection of transformed worms *via* the *pha-1* marker.

To generate the *ufm-1* deletion mutant (WWU1002 *(ufm-1(eva202)III))*, we employed CRISPR/Cas9-based genome editing, using *dpy-10* as a co-CRISPR marker (35). The sgRNA targeting *ufm-1* was cloned into pRB1017 (Addgene) and this plasmid, along with pJA58, pDD162 (Addgene) and a repair template, was injected into WT worms. To generate the *ufm-1* deletion mutant, we screened the F1 generation for coedits *via* PCR (primers are listed in Table S1) and BamHI restriction digest. The introduction of a BamHI restriction site into the repair template allowed genotyping of worms. Candidates were subsequently validated by Sanger sequencing and outcrossed to eliminate potential background mutations. A detailed schematic of the CRISPR/Cas9 strategy is provided in Fig. S2 and all related molecular reagents (PCR primers, sgRNA, and ssOligo donor sequences) are listed in Table S1.

The OH441 strain, which exhibits pan-neuronal GFP expression, and the LX929 strain, which shows cholinergic neuronal GFP expression, were crossed with the *ufm-1* deletion mutant strain WWU1002 to generate the strains WWU1009 *[ufm-1(eva202)III; otIs45 unc-119::gfp]* and the strain WWU1019 [*ufm-1(eva202)III; vsls48*]. Both strains were then used to investigate how the deletion of *ufm-1* affects neuronal function and integrity throughout the lifespan of *C. elegans*.

A rescue construct was generated by cloning the genomic region of *ufm-1*, including its native promoter, into the pPD49_83 vector. The plasmid was coinjected with a *myo-2::gfp* coinjection marker into the *ufm-1* deletion mutant to generate extrachromosomal arrays. Transgenic lines, designated as WWU1018 *(evaEx211 [ufm-1p::ufm-1 + myo-2p::gfp; ufm-1(eva202)III]),* were established and confirmed by PCR. Lifespan, development, reproduction, motility, and stress resistance assays were subsequently performed to assess the phenotypic rescue of the *ufm-1* deletion mutant.

*C. eleg*ans was cultured on nematode growth medium (NGM) plates seeded with *Escherichia coli* OP50 at 20 °C or 25 °C under standard conditions (66). To synchronize populations, hypochlorite treatment was performed to induce synchronous hatching of eggs from gravid adults (67).

### RNAi treatments

RNAi treatments were performed by feeding, following standard procedures (68). HT115 *E. coli* strains containing RNAi clones in the L4440 vector or an empty vector were cultured on NGM plates supplemented with ampicillin and IPTG to induce dsRNA expression. Synchronized L1 larvae were then transferred to these RNAi plates and incubated under standard conditions for subsequent analysis.

### Gene expression analyses under normal and stress conditions

To investigate the spatiotemporal expression pattern of *ufm-1*, the transgenic strain WWU1001 was used. After synchronization, worms were imaged at different developmental stages using a laser-scanning microscope with images captured every 24 h for three consecutive days.

To assess stress-induced expression, GFP fluorescence was analyzed following exposure of L4 larvae or young adult worms to various stress conditions: heat shock (30 °C, 4 h), 0.1 mM cadmium chloride (4 h), 0.1 mM juglone (4 h), 3 mM and 5 mM DTT (4 h), 3 μg/ml and 5 μg/ml tunicamycin (4 h), and 10 μM, 50 μM, and 100 μM rapamycin (4 h).

For imaging, worms were mounted on a slide with a 1% agarose pad and immobilized using levamisole (100 μM). Each stress condition was tested in triplicates and image quantification was performed using ImageJ software to assess the GFP expression levels across different conditions.

### DAPI staining

To assess the subcellular localization of UFM-1, we examined a transgenic *C. elegans* line expressing GFP-tagged UFM-1 under its endogenous promoter. For nuclear visualization, worms were fixed and stained with DAPI. Fixation was performed using an ethanol-based protocol that preserves both GFP fluorescence and subcellular protein distribution (69). Fluorescence imaging was performed using a standard epifluorescence microscope. Colocalization with DAPI was used to investigate nuclear accumulation, as indicated by merged GFP and DAPI signals.

### Life history traits and stress resistance assays

Morphological analysis of the *ufm-1* loss-of-function mutant was performed on synchronized young adult worms to evaluate structural differences in *ufm-1* deletion mutants compared to WT worms. Differential interference contrast microscopy was used to examine body morphology, overall structure, and gonads, with a focus on abnormalities in gonadal and oocyte development.

To assess life-history traits and stress resilience, several assays were performed using synchronized L4 larvae.

For lifespan assays, 10 worms were transferred to one small NGM plate seeded with *E. coli* OP50 and moved to a new plate daily while monitoring survival *via* touch response. The total number of living and dead animals was recorded.

For reproduction assays, L4 larvae were placed on small plates and transferred to a fresh plate every 24 h for nine to 10 days. The progeny of each worm was counted after 24 h of incubation on the old plate. In addition, the development of offspring was monitored over time. three to five adult worms were placed on a small plate and removed after laying 10 to 15 eggs. The developmental stages of these eggs were evaluated after 24 h, 48 h, and 72 h.

Stress resistance assays were performed using synchronized young adult worms. Worms were placed on small NGM plates seeded with *E. coli* OP50 and exposed to various stressors: 3 mg/ml CdCl_2_, 400 mM NaCl, 0.2 mM juglone, 11 mM DTT, and 9 mM TCEP. Survival was determined by touch stimulus after 18 to 24 h.

### Behavioral assays

Exploration behavior was assessed by placing young adult worms separately on medium plates fully seeded with OP50. After an 18 h incubation, exploration of the worms was quantified using a grid system (70, 71).

To assess radial locomotion, 10 young adult worms were placed at the center of an NGM plate without bacteria. After 30 min, the position of each worm was marked and the distance from the center was measured to evaluate their movement capacity (72).

Swimming behavior was analyzed using the CeleST software (38), which facilitates automated quantification of *C. elegans* motility in liquid medium. Adult worms were recorded while swimming and videos were processed frame-by-frame to capture detailed motion characteristics. CeleST computes multiple swim behavior parameters that provide insights into both the intensity and coordination of swim pattern. Data from multiple independent replicates were collected to assess any phenotype differences.

### UPR reporter assay procedures

The *ufm-1* deletion allele *ufm-1(eva202)* was crossed into the reporter strains SJ4005 (*hsp-4::gfp*, a marker for UPR_ER_) and SJ4100 (*hsp-6p::gfp* + *lin-15(+)*, a marker for UPR_mito_). Homozygous *ufm-1; hsp-4::gfp* and *ufm-1; hsp-6::gfp* double mutants were generated and verified by PCR and fluorescence microscopy. L4-stage worms from these crossed strains, as well as the respective reporter-only controls, were mounted on 1% agar pads and immobilized with levamisole for imaging under identical acquisition settings.

Whole-animal GFP fluorescence was quantified using ImageJ to measure mean intensity. Data were collected from three independent biological replicates, with each replicate containing ≥10 worms per condition.

### *C. elegans* models of proteotoxicity

Worm strains expressing Aβ (CL2006, *dvIs2[pCL12(unc-54p::human Aβ) + rol-6(su1006)])*, α-syn (UA49, *baIn2[unc-54p::α-syn::gfp, rol-6(su100*6)]) or a fragment of mutant human Huntington protein (EAK103, *eeeIs2[unc-54p::Htt513(Q128)::yfp::unc-45 3′UTR])* were used to assess neurodegeneration after RNAi-mediated knockdown of *ufm-1*.

Paralysis in CL2006 worms results from Aβ expression and accumulation in body wall muscle. Worms were maintained at 15 °C and heat-stressed at 37 °C for 1 h to accelerate paralysis. Paralysis was scored as a restriction of movement to the head region only or a complete lack of movement in response to gentle touch with a worm pick. Worms that did not move but showed no signs of pharyngeal pumping were considered dead and excluded from the analysis.

In UA49 worms, α-synuclein aggregate size and number were quantified 2 to 3 days posthatching as described by Starr *et al.* (73). Briefly, the head region of young adult worms was imaged by laser scanning microscopy and aggregate size and count was analyzed using ImageJ.

EAK103 worms express a YFP-tagged, polyQ-expanded fragment of human Huntingtin (513 amino acids) in body wall muscles. PolyQ aggregation was quantified by measuring YFP fluorescence intensity and aggregate size and count using fluorescence microscopy and ImageJ.

### Assessment of sensory neuron function *via* dye filling assay

A dye filling assay was used to assess the integrity of specific sensory neurons in *C. elegans*, particularly amphid and phasmid neurons, which contain ciliated structures essential for movement and environmental sensing (74, 75, 76). For this purpose, the fluorescent lipophilic dye DiO was used, which selectively fills intact ciliated neurons. Age-synchronized adult WT and *ufm-1* mutant were imaged at distinct days of adulthood (days 1, 6, 9, and 12). Worms were incubated in a DiO solution, allowing the dye to be taken up by the sensory cilia. Following incubation, they were examined under fluorescence microscopy to assess neuron staining, using protocols adapted from (77). Dye uptake was quantified using ImageJ to analyze fluorescent area per head region.

### Analysis of neuronal integrity during aging

To assess the role of UFM-1 in maintaining neuronal integrity during aging, the pan-neuronal and cholinergic neuron-specific GFP-expressing strains OH441 and LX292 were crossed into the *ufm-1* mutant background. Age-synchronized adult populations of the resulting crosses were imaged at distinct days of adulthood (days 1, 6, 9, and 12). At each time point, worms were immobilized on 1% agarose pads using 30 mM levamisole as an anesthetic. High-resolution images of the pan-neuronal and cholinergic GFP signals were acquired using a laser scanning microscope under identical acquisition parameters. Neuronal integrity was assessed by quantifying overall GFP intensity or counting morphological defects, such as neurite breaks and beading, within a defined region of the nervous system, with the experimenter blinded to the genotype throughout the analysis.

### SDS-PAGE and Western blot

Worm lysis was performed by grinding worm pellets with a plastic pestle in Laemmli buffer. Whole worm extract was analyzed by 12.5% SDS-PAGE. Proteins were revealed by Coomassie Blue staining and Western blot analysis using a rabbit polyclonal antibody against UFM-1 at a dilution of 1:1000. A monoclonal anti-rabbit antibody from donkey with alkaline phosphatase was used as a second antibody at a dilution of 1:10,000.

## Data availability

All data are contained within the article.

## Supporting information

This article contains supporting information.

## Conflict of interest

The authors declare that they have no conflicts of interest with the contents of this article.
