# Supplementary figures and images for: Exploring the organismal role of UFMylation in development, stress resilience, and neurological function in Caenorhabditis elegans
Source: J Biol Chem. 2026 Jun 12;302(8):113247. doi: 10.1016/j.jbc.2026.113247 (PMC13351140; doi:10.1016/j.jbc.2026.113247)

**A**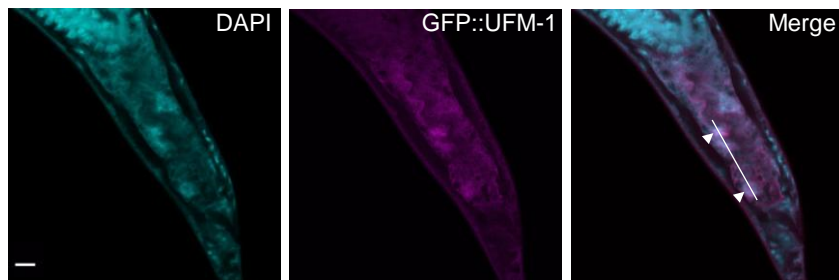**B**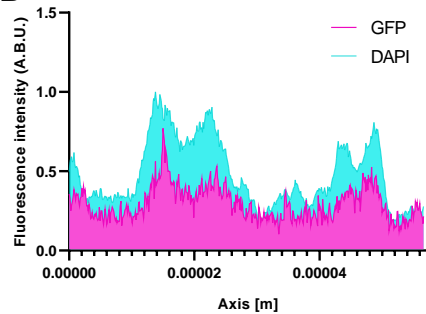**C**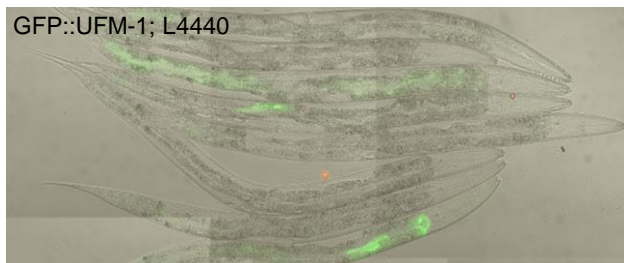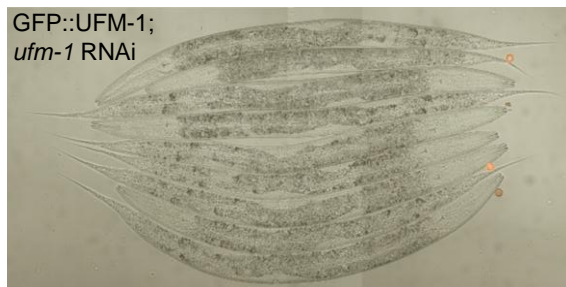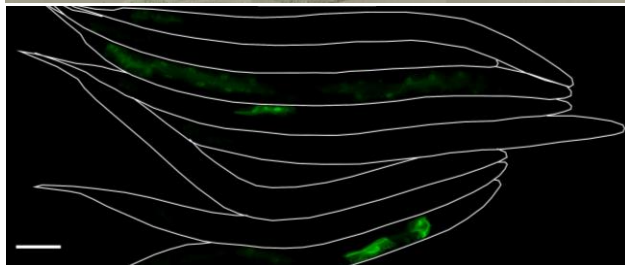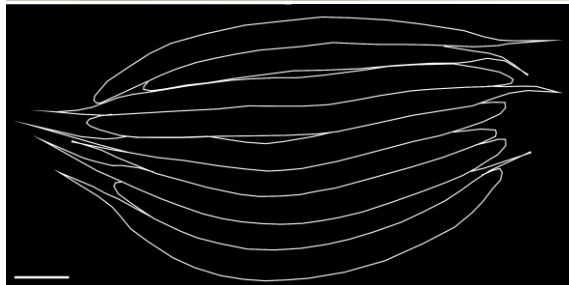

Supplement: Figure S1 [file mmc1.pdf]

**A**

Fluorescence intensity (A.B.U.)

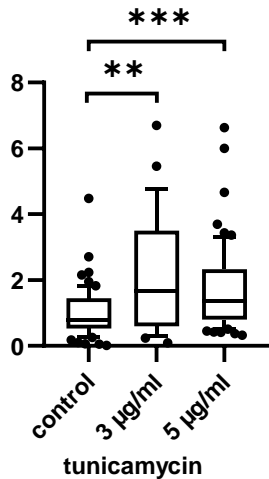**B**

Fluorescence intensity (A.B.U.)

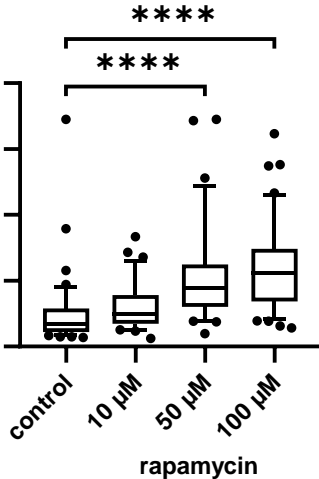**C**

Fluorescence intensity (A.B.U.)

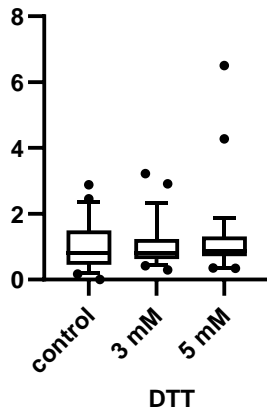

Supplement: Figure S2 [file mmc2.pdf]

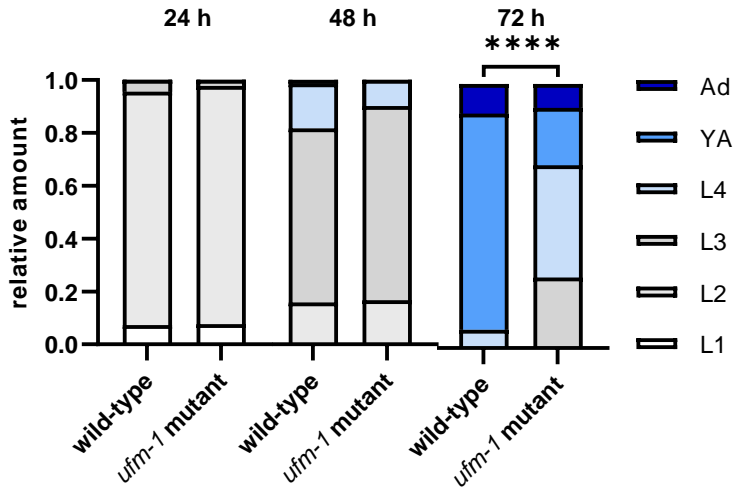

Supplement: Figure S4 [file mmc4.pdf]

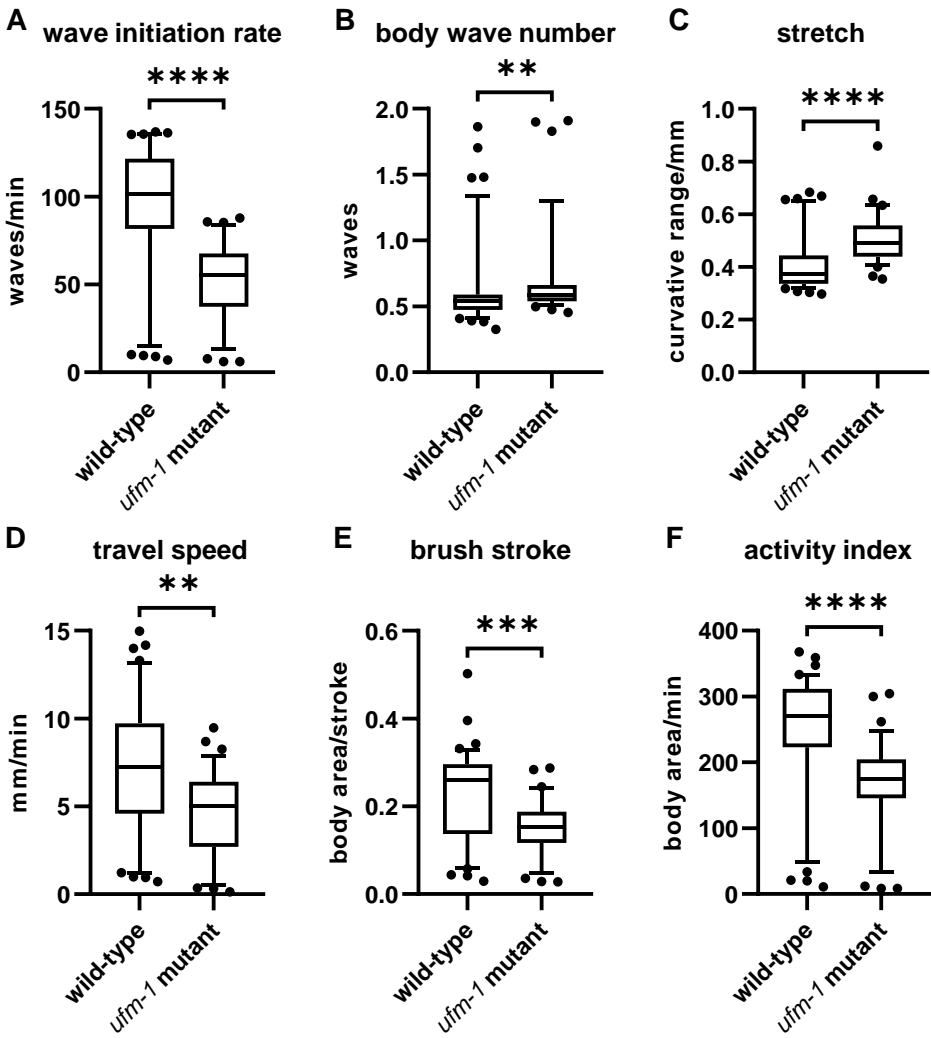

Supplement: Figure S5 [file mmc5.pdf]

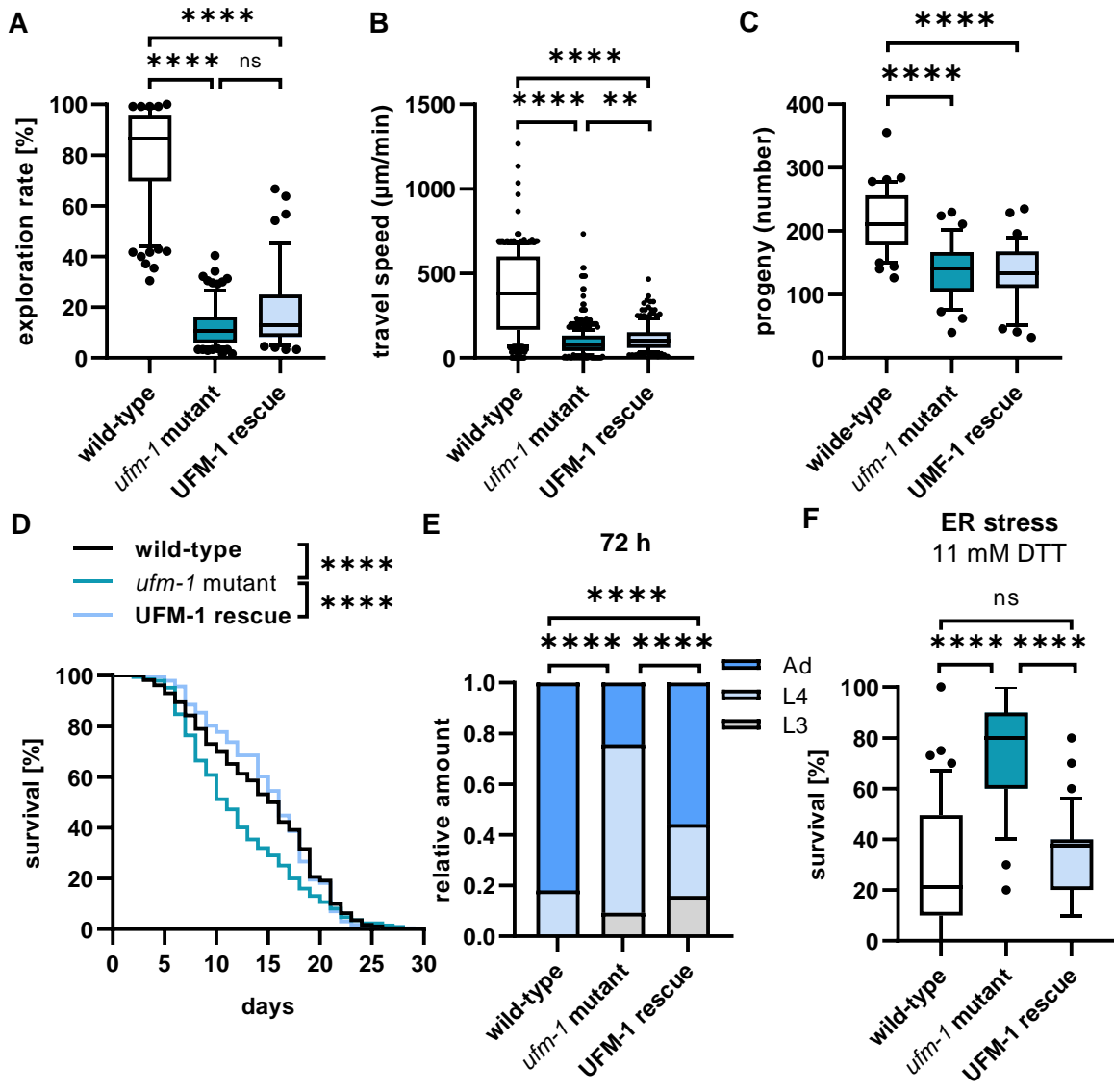

Supplement: Figure S6 [file mmc6.pdf]
