# Supplementary material for: Exploring the organismal role of UFMylation in development, stress resilience, and neurological function in Caenorhabditis elegans
Source: J Biol Chem. 2026 Jun 12;302(8):113247. doi: 10.1016/j.jbc.2026.113247 (PMC13351140; doi:10.1016/j.jbc.2026.113247)
Supplement: Figure S3 [file mmc3.pdf]

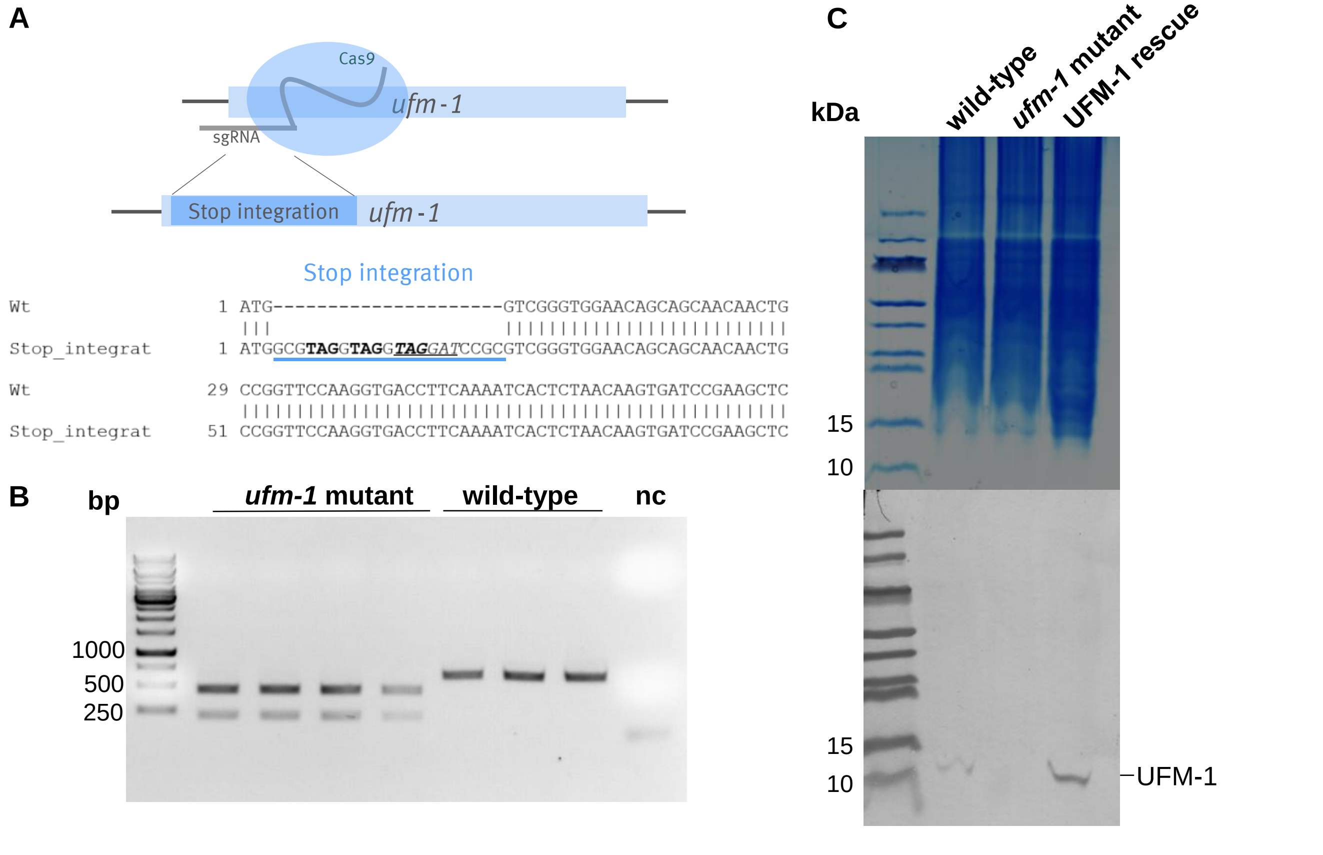

A
Cas9
ufm-1
sgRNA
Stop integration
ufm-1
Stop integration
Wt 1 ATG--------------------GTCGGGTGGAACAGCAGCAACAACTG
Stop_integrat 1 ATGGCGTAGGTAGGTAGGATCCGCGTCGGGTGGAACAGCAGCAACAACTG
Wt 29 CCGGTTCCAAGGTGACCTTCAAAATCACTCTAACAAGTGATCCGAAGCTC
Stop_integrat 51 CCGGTTCCAAGGTGACCTTCAAAATCACTCTAACAAGTGATCCGAAGCTC
B
bp
ufm-1 mutant
wild-type
nc
1000
500
250
C
kDa
wild-type
ufm-1 mutant
UFM-1 rescue
15
10
15
10
UFM-1
