# Supplementary material for: Exploring the organismal role of UFMylation in development, stress resilience, and neurological function in Caenorhabditis elegans
Source: J Biol Chem. 2026 Jun 12;302(8):113247. doi: 10.1016/j.jbc.2026.113247 (PMC13351140; doi:10.1016/j.jbc.2026.113247)
Supplement: Supporting information [file mmc7.docx]

**Supporting Information**

**Tables**

**Table S1.** Oligonucleotides used for sequencing, CRISPR/Cas9-mediated knockout and rescue of *ufm-1*.

| Name | Sequence | Tm [°C] | Application |
| --- | --- | --- | --- |
| Genotyping of *C. elegans* deletion mutants | | | |
| Ufm-1 Seq S | GCCGAGTTTTGTAAGAGACACTTGGC | 67.9 | Genotyping and sequencing of *ufm-1* |
| Ufm-1 Seq AS | GCGAGTTTAGGATAGAAATGCACAGAGAC | 69.1 | Genotyping and sequencing of *ufm-1* |
| Generation of sgRNA and repair template | | | |
| BsaI sgRNA Ufm-1 S | TCTTGGATTTTCAGGAATGTCGGG | - | Generation of the insert for pRB1017 |
| BsaI sgRNA Ufm-1 AS | AAACCCCGACATTCCTGAAAATCC | - | Generation of the insert for pRB1017 |
| *ufm-1* stop repair | actggacgattattattgattttatgcgagagattttcaggaATGGCGTAGGTAGGTAGGATCCGCGtcgggtggaacagcagcaacaactgccggttccaaggtgaccttc |  | Repair template for CRISPR/Cas9, mediating stop codon integration to *ufm-1* |
| Cloning primers | | | |
| IFpPD49_83 *ufm-1p::ufm-1* HindIII S | CCATGATTACGCCAAGCTTGTGACAAAATAAACGAAACTTTTTTC | 65.5 | Cloning of *ufm-1p::ufm-1* in pPD49_83 |
| IFpPD49_83 *ufm-1p::ufm-1* BamHI AS | CCAATCCCGGGGATCCTCCTGAAAATCTCTCGCAT | 69.1 | Cloning of *ufm-1p::ufm-1* in pPD49_83 |

**Figure legends**

**Figure S1. Subcellular localization of GFP::UFM-1.** (A) Representative image showing the distribution of GFP-tagged UFM-1 (pink) in intestinal cells. The signal is present in the cytosol and also localizes at the nuclei, as indicated by its colocalization with DAPI staining (blue). White arrowheads in the merged panel highlight representative nuclei where the signals overlap (scale bar 10 µm). (B) The subcellular fluorescence intensity was analyzed by performing a virtual line scan at the region of interest (white line). (C) Shows representative images GFP::UFM-1 on control plates (L4440) and knockdown via *ufm-1* RNAi.

**Figure S2. GFP::UFM-1 expression in *C. elegans* under different concentrations of stressors.** The stress-responsive expression of UFM-1 was assed using *C. elegans* expressing GFP-tagged UFM-1 under its native promoter. Worms were exposed to different concentrations of stressors for 4 hours (A) tunicamycin (3 µg/ml (p=0.004) and 5 µg/ml (p=0.0005)), (B) rapamycin (10, 50 (p<0.0001) and 100 µM (p<0.0001)) and (C) DTT (3 and 5 mM). Fluorescence intensity was analyzed using ImageJ software across three independent trials. Statistical significance relative to control was assessed using Kruskal-Wallis test. Box and whisker plots show the median (central line), 25^th^ to 75^th^ percentiles (boxes), and 10^th^ to 90^th^ percentiles (whiskers). Values outside this range are displayed as individual points.

**Figure S3. Strategy for the CRISPR/Cas9-mediated knockout of *ufm-1*.** (A) A target site near the 5′ end of the gene was selected to create a frameshift and a repair template was designed to introduce early stop codons (bold) and a novel BamHI site (italics underlined) for genotyping. The sequence alignment confirms the successful edit. (B) Genotypic validation by PCR and restriction digest. Genomic DNA from wild-type and the mutant strain *WWU1002, ufm-1(eva202)* was amplified and digested with BamHI. (c) Protein-level validation. Coomassie staining (upper panel) shows total protein. Western blot analysis (lower panel) of lysates from wild-type, *ufm-1* mutant and the rescue strain WWU1018, *evaEx211 [ufm-1p::ufm-1 + myo-2p::gfp; ufm-1(eva202)*]. The knockout and subsequent rescue were confirmed at the protein level by western blot analysis using an anti-UFM-1 antibody, which showed the absence of the protein in the mutant and its restoration in the rescue strain. All oligonucleotides used for this procedure are listed in Table S1.

**Figure S4. Larval development of *ufm-1* mutant.** Animals were scored for larval stage distribution (L1-4, larval stage 1-4; YA, young adult; Ad, adult) and compared to wild-type at 24 h, 48 h and 72 h (*wt*, n=254; *ufm-1* mutant, n=109, Sidak’s test).

**Figure S5. CeleST analysis of locomotion parameters in *ufm-1* mutants compared to wild-type.** CeleST was used to analyze video-based locomotion parameters for wild-type (n=41) and *ufm-1* mutants (n=37) in liquid media (Mann-Whitney U test). (A) The wave initiation rate, reflecting the frequency of body wave generation per minute, was significantly reduced in *ufm-1* mutants, indicating diminished swimming activity (p<0.0001). (B) The body wave number was increased, suggesting a shift towards crawling-like behavior and impaired coordination between swimming and crawling (p=0.0045). (C) Stretch, defined as the maximum curvature range during strokes, was significantly higher in the *ufm-1* mutants, indicating altered bending dynamics (p<0.0001). (D) Travel speed, which measures the distance covered per minute, was notably lower in *ufm-1* mutants, pointing to reduced locomotor efficiency (p=0.0010). (E) The brush stroke area, reflecting the body’s flexion during strokes, was significantly reduced in mutants, indicating weaker swimming (p=0.0004). (F) The activity index, which measures the total body area moved per minute, was significantly decreased in *ufm-1* mutants (p<0.0001). Box and whisker plots show the median (central line), 25^th^ to 75^th^ percentiles (boxes), and 10^th^ to 90^th^ percentiles (whiskers). Values outside this range are displayed as individual points.

**Figure S6. Partial phenotypic rescue in the *ufm-1* rescue strain.** (A-D) The *ufm-1* deletion mutant WWU1002, *ufm-1(eva202)* was rescued with an extrachromosomal array expressing the wild-type *ufm-1* genomic sequence under its native promoter, creating strain WWU1002; Ex[*ufm-1p::ufm-1 +myo-2p::gfp*]. (A) Exploration behavior remained impaired in the rescue strain compared to wild-type (*wt*: n≥225; *ufm-1* mutant: n≥225 (p<0.0001); rescue: n≥225 (p<0.0001)). (B) Radial locomotion showed significant but partial restoration (*wt*: n≥45; *ufm-1* mutant: n≥45 (p<0.0001); rescue: n≥45 (**** p<0.0001; **p=0.0081)). (C) Reproduction was not restored in the rescue strain (*wt*: n=40; *ufm-1* mutant: n=36 (p<0.0001); rescue: n=39 (p<0.0001)) but (D) lifespan was fully rescued to wild-type levels (*wt*: n≥250; *ufm-1* mutant: n≥250 (p<0.0001); rescue: n≥250 (p<0.0001)) and (E) development of L4 larvae after 72 h was restored (*wt*: n=228; *ufm-1* mutant: n=234 (p<0.0001); rescue: n=380 (p<0.0001)). (F) Stress tolerance was completely restored (*wt*: n≥300; *ufm-1* mutant: n≥300 (p<0.0001); rescue: n≥300 (p<0.0001)). Data were analyzed by Dunn's method (A, B, D, F), Kaplan-Meier log-rank test (D) and Tukey’s multiple comparisons test (C, E). Box and whisker plots show the median (central line), 25^th^ to 75^th^ percentiles (boxes), and 10^th^ to 90^th^ percentiles (whiskers). Values outside this range are displayed as individual points.
